# Deciphering the constraints of pure bacterial strains for the complete catabolism of sulfamethoxazole: A proteomic and kinetic study

**DOI:** 10.1007/s10532-025-10211-8

**Published:** 2025-11-05

**Authors:** Ana P. Lopez Gordillo, Alba Trueba-Santiso, Kilian E. C. Smith, Andreas Schäffer, Juan M. Lema

**Affiliations:** 1 Institute for Environmental Research, RWTH Aachen University, Worringerweg 1, 52074 Aachen, Germany; 2 CRETUS, Department of Chemical Engineering, Universidade de Santiago de Compostela, 15782 Santiago de Compostela, Galicia, Spain; 3 Environmental Chemistry, Department of Water, Environment, Construction and Safety, University of Applied Sciences Magdeburg-Stendal, Breitscheidstraße 2, 39114 Magdeburg, Germany

**Keywords:** Trace organic compounds, Direct metabolism, SadABC, Dihydropteroate synthase, *Microbacterium* sp. BR1

## Abstract

Organic micropollutants (OMP) pose a significant environmental challenge, and microbial degradation research typically involves monitoring parent compound depletion and metabolite production. Previous studies on the antibiotic sulfamethoxazole (SMX) have shown its incomplete biotransformation by either mixed microbial communities or acclimated pure bacterial across various concentrations. However, the mechanisms behind this incomplete degradation and its relationship with the enzymatic capacities and expressions at environmentally relevant concentrations remain unclear. Therefore, this study investigated the biotransformation of SMX and the variations in the proteome at low µg L^−1^ concentrations using acclimated *Microbacterium* sp. BR1 as the bacterial degrader. Results show an incomplete depletion of the SMX and accumulation of the metabolite 3-amino-5-methylisoxazole (3A5MI). All test concentrations triggered the expression of the sulfonamide degrading enzymes (SadAB) and the modified target enzyme (Sul). Analysis of the functional proteins revealed increased cellular regulation and confirmed the bacterial strain's continued activity throughout the experiment. This suggests that at low SMX concentrations, even a highly sensitive and metabolically active strain may still require complementary enzymatic machinery to degrade potentially inhibitory metabolites. Thus, this study provides important insights into the persistence of SMX and reveals the complexities of antibiotic biodegradation at environmentally relevant concentrations, highlighting the need for comprehensive understanding of enzymatic mechanisms in micropollutant remediation strategies.

**Supplementary Information:**

The online version contains supplementary material available at 10.1007/s10532-025-10211-8.

## Introduction

Organic micropollutants (OMP) are contaminants that represent a hazard to the environment and human health despite their presence in the environment at typically low concentrations of micrograms to nanograms per litre. Biological treatment using microbial biotransformation is widely used for OMP conversion, usually into less dangerous compounds (Fenner et al. 2021). In some cases, however, biotransformation forms metabolites, which can be less biodegradable and even more toxic than the original parent OMP molecule. Consequently, investigations into the biotransformation of OMP should include quantifying both depletion in the parent OMP supplemented by analysis of the metabolites that are formed (Reis et al. 2020a).

Studies into the biotransformation mechanisms for antibiotic removal are of particular interest due to their potential to unleash the development of bacterial resistance (Baquero et al. 2008; Manaia et al. 2016). From within this group, sulfonamides have been widely studied (Reis et al. 2020b), with sulfamethazine (SMZ), sulfadiazine (SDZ), sulfapyridine (SPY), and sulfamethoxazole (SMX) being among the most investigated compounds (Nunes et al. 2020).

SMX biotransformation may occur through direct metabolism or co-metabolism. SMX direct metabolism involves the breakdown of the parent compound and the production of numerous metabolites. These include 4-aminophenol, hydroquinone, 3-amino-5-methylisoxazole (3A5MI), 1,2,4-trihydroxybenzene, 4-aminothiophenol, 4-amino-benzesulfonic, 4-amino-benzesulfonamide, aniline, sulfanilamide, 4-aminobenzenesulfonamide and sulfanilic acid (Bouju et al. 2012; Jiang et al. 2014; Reis et al. 2014; Mao et al. 2018; Mulla et al. 2018; Shi et al. 2025). In contrast, SMX co-metabolism involves simple reactions that only modify the parent compound and enhance or partially keep its toxicity (Majewsky et al. 2014). Examples of single transformation reactions accomplished co-metabolically by bacteria in soil and water and axenic cultures (Nunes et al. 2020) in the presence of SMX include acetylation, hydroxylation, or nitration at the *para* amino group. Therefore, reported biotransformation products formed co-metabolically comprise N^4^-acetylsulfamethoxazole (Ac-SMX) (Reis et al. 2018), N^4^-hydroxy-acteylsulfamethoxazole (OH-Ac-SMX) (Larcher and Yargeau 2011), 4-hydroxyl-N-(5-methyl-1,2-oxazole-3-yl)benzene-1-sulfonamide (4-OH-SMX) (Gauthier et al. 2010) and 4-nitro-sulfamethoxazole (NO_2_-SMX) (Kassotaki et al. 2016).

Occasionally, SMX and its metabolites undergo incomplete biological removal. Possible causes for this may be linked to the toxicity of the formed metabolites, their back transformation to the parent OMP, or a retro-inhibition (Gonzalez-Gil et al. 2018; Rios-Miguel et al. 2023). Some examples include the toxic metabolites NO_2_-SMX and 4-OH-SMX, which inhibit *Vibrio fischeri* growth (Majewsky et al. 2014). Also, some SMX metabolites formed in axenic cultures and which are initially stable, can be back-transformed to SMX when present in complex environments (Chen and Xie 2018). Such back transformation has been described for Ac-SMX (Radke et al. 2009) and 4-NO_2_-SMX, the latter under nitrate starvation (Nödler et al. 2012).

In addition to analysis of parent SMX and its metabolites, proteomics allows a deeper understanding of the (in)complete degradation of SMX, as it provides information on the degradation mechanism of xenobiotic removal and the bacterial metabolic response (Zhang et al. 2024). Proteomic studies on SMX biodegradation may thus include analysis of the proteins and resistance mechanisms involved in sulfonamide biodegradation, such as sulfonamide monooxygenases (Sad cluster) (Reis et al. 2019), modifications of the enzyme dihydropteroate synthase (DHPS) (*fol*P and Sul genes: *sul*1-*sul*4) (Sánchez-Osuna et al. 2018), the enzyme arylamine N-acetyltransferase (NAT) (Cribb et al. 1993; Kagaya et al. 2012) and multi-drug efflux pumps systems (MexAB-OprM and *smeDEF)* (Blair et al. 2014; Sánchez and Martínez 2015).

Several investigations into SMX biotransformation have been conducted at mg L^−1^ concentrations (Herzog et al. 2013; Ricken et al. 2013; Jiang et al. 2014; Reis et al. 2014; Wang and Wang 2018). However, the underlying biotransformation may differ at lower concentrations (Knapp and Bromley-Challoner 2008; van Bergen et al. 2021; Yan et al. 2023), which are more representative of those found in the environment. For instance, SMX environmental concentrations lie within ranges from 3.6 × 10^–4^ µg SMX L^−1^ to 5.32 µg SMX L^−1^ in surface water (Zhang et al. 2012; Matongo et al. 2015), from 6.52 × 10^–2^ µg SMX L^−1^ to 12.85 µg SMX L^−1^ in the effluent of wastewater treatment plants (Zhou et al. 2013; Dinh et al. 2017), and from 1.01 × 10^–3^ µg SMX L^−1^ to 2.51 × 10^–1^ µg SMX L^−1^ in groundwater (Archundia et al. 2017; Gray et al. 2020). Moreover, recent research into SMX biotransformation lacks a comprehensive approach that integrates a mechanistic insight and enzymatic regulation at the low µg L^−1^ level (Song et al. 2021; Liu et al. 2022; Qi et al. 2022; Wang et al. 2023; He et al. 2024).

To address the need for information on antibiotics and their transformation products at environmentally relevant concentrations (Yang et al. 2021), the present study uses *Microbacterium* sp. BR1 as a degrader model to investigate the mechanisms behind the incomplete removal of low concentrations of SMX (Lopez Gordillo et al. 2024). This novel approach integrates mass spectrometric analyses of SMX and its metabolites with measurements of the proteome profile of the bacterial sulfonamide degrader.

## Materials and methods

### SMX solutions

SMX (purity 98%, Sigma Aldrich) was used to prepare a stock solution of 2 × 10^4^ µg SMX L^−1^ in Milli Q water. Two solutions of SMX were prepared in phosphate saline buffer (PBS) pH 7.4 through dilutions of this stock solution, and these were then sterilised at 121°C for 15 min. These solutions were used to spike the corresponding reactors to give SMX starting concentrations of 20 µg L^−1^ and 12 µg L^−1^. These concentrations were selected based on their environmental relevance and to complement results from an analogous study describing an incomplete SMX mineralisation (Lopez Gordillo et al. 2024).

### Biomass acclimation and production

*Microbacterium* sp. BR1 was selected as a suitable bacterial degrader of SMX due to its capability to use SMX as the only source of carbon and energy, and its known SMX degrading enzymes (Ricken et al. 2017). Dr. Boris Kolvenbach from the Institute for Ecopreneurship, University of Applied Sciences and Arts (Northwestern Switzerland) provided a sample of this pure strain. Sterile standard media 1 at 25% (Carl Roth) containing 2.53 × 10^5^ µg SMX L^−1^ was used for these cultures (Ricken et al. 2015). Culture flasks were kept in darkness and incubated at 28 °C with an agitation of 140 rpm. When the optical density (OD_600_) reached a value of 1.4, the cultures were centrifuged at 7000 g and 4 °C for 20 min and washed with cold 0.85% sodium chloride (NaCl). After two washes, the bacterial pellets were homogeneously resuspended in 0.85% NaCl and 20% glycerol. This resuspension was split into several aliquots and stored at -80°C until used in the biotransformation tests. This acclimation to SMX aimed to trigger the enzymes involved in the catabolism, which is necessary in such short biotransformation tests.

### Biotransformation tests

Batch experiments were performed for each of the two SMX test concentrations. In each batch, the reactors consisted of 50 mL amber glass bottles filled with 26 mL of sterile PBS (n = 3 biological replicates for each time point). To avoid potential contamination from used laboratory glassware, only new glass bottles and lids were used for the test and were autoclaved before their use. One mL of the appropriate sterile SMX spiking solution was added to each bottle to obtain the required initial SMX concentration. Biotransformation assays were started by adding 1 mL of the thawed *Microbacterium* sp. BR1 aliquots to each reactor. The bacterial density at the start was determined by streaking serial dilutions of the aliquot on agar plates and calculating the colony-forming units (CFU), which was 209.8 ± 0.34 × 10^6^ CFU mL^−1^. To exactly reproduce the conditions of a separate study aimed at measuring SMX mineralisation (Lopez Gordillo et al. 2024), an insert preloaded with 1 M potassium hydroxide (KOH) was also added to the bottles before their closure with a screw cap. Abiotic controls consisted of reactors containing SMX without bacteria, and reactors containing SMX with inactive (autoclaved) bacteria. The reactors were kept at 22 °C under horizontal agitation at 140 rpm in the dark.

Each biotransformation test lasted 24 h, and there were four sampling times: 2 h, 4 h, 8 h, and 24 h. Triplicate reactors from each test concentration were sacrificed and opened inside the sterile bench for each time point. The KOH and the insert were carefully withdrawn and disposed of (note that these were only added to reproduce the set-up used for the mineralisation experiment). The reaction medium was mixed before being transferred to 50 mL sterile tubes and centrifuged at 2200 g and 4°C for 35 min. The supernatant was separated from the bacterial pellet, frozen and stored for further analysis of SMX and the metabolite 3A5MI using Liquid Chromatography coupled to Mass Spectrometry (LC–MS/MS). The bacterial pellet was washed twice with ice-cold 0.85% NaCl, centrifuged using the same conditions as above, and the supernatant discarded each time. Finally, the bacterial cells were resuspended in 2 mL of cold 0.85% NaCl and frozen until their preparation for proteome analysis.

### Analysis of SMX and 3A5MI

Defrosted supernatants of the tests performed at 20 µg L^−1^ and 12 µg L^−1^ were centrifuged again at 6000 g and 4 °C for 20 min before their analysis. A SMX standard addition approach was used to counteract any matrix effects. The samples were analysed as follows: a 100 µL sample volume was injected into an ultra-high-performance liquid chromatograph (UHPLC ELUTE, Bruker). Sample up-concentration was achieved via online extraction (OLE) before chromatographic separation on a C18 column (Intensity solo, Bruker). In the MS (timsTOF PRO, Bruker), the analytes were ionised with electrospray ionisation (ESI) in positive mode and fragmented with a broadband collision-induced dissociation (bbCID). A triple quadrupole (QQQ) and a Quadrupole Time-of-Flight (QTOF) analysers were used for the screening. Details on the analytical method are given in the online resource Text S1 and online resource Table S1. The analytical limit of quantification (LOQ) was 0.5 µg SMX L^−1^. SMX depletion data was fitted using a one phase decay model, with the software GraphPad Prism 5, to define whether a plateau on SMX concentration could be distinguished during the test.

### Proteome analysis

Samples from the 20 µg L^−1^ and 12 µg L^−1^ reactors were pretreated as follows: a bacterial pellet replicate from each sampling point was thawed and centrifuged at 6000 g and 4°C for 20 min, followed by disposal of the 0.85% NaCl supernatant. Proteins were extracted using the method described in the study by Kennes et al. (Kennes-Veiga et al. 2022). For this, protein release and denaturation were achieved through cell lysis using digestion with 1% of sodium dodecyl sulfate (SDS) pH 7.5 at 90°C for 20 min and four cycles of mechanical disruption, each with a duration of 3 min, by beating with glass beads using a cell disruptor (Scientific Industries, USA). After centrifugation at 1200 g at 4°C for 20 min, the proteins contained in the supernatant were transferred to microcentrifuge tubes and precipitated with cold acetone by incubation at -20°C. Acetone was removed after centrifugation at 10,600 g and 4°C for 10 min, and the proteins were resuspended in molecular-grade water and acetone (1:4 v:v). After another incubation step at -20°C, the samples were centrifuged as above, and the supernatant was discarded. Finally, the extracted proteins were resuspended in molecular-grade water and frozen.

Total protein concentrations were quantified using the Pierce bicinchoninic acid protein assay kit (BCA, Thermo Scientific) (Online resource Table S2). An SDS-PAGE electrophoresis with a NuPAGE gel (4–12% Bis–Tris acrylamide, Thermo Fischer) was performed under denaturing conditions to verify any protein degradation in the extracts. The electrophoresis was run with aliquots containing 10 µg protein, and the gel was stained with a standard Coomassie protocol (Online resource Fig. S1).

Frozen protein extracts were digested with trypsin and desalted prior to their analysis using mass spectrometry. For the shotgun proteomic analysis (Zhang et al. 2013), 200 ng of digestate from each sample were injected into a nano UHPLC chromatograph (nano ELUTE, Bruker) for normalization and to ensure comparability between the samples. The nano ELUTE was equipped with an Aurora analytical column (C18, 250 × 0.075 mm, 1.6 μm, 120 Å, IonOpticks) coupled to a time-QTOF Pro analyser (Bruker). The mobile phases included 0.1% formic acid in milliQ water (A) and 0.1% formic acid in acetonitrile (B). The analysis time per sample or blank lasted 40 min with a gradient flow between both mobile phases. Any carry-over was avoided via the analysis of blanks between samples. The separation of the peptides was followed by their fragmentation with a collision-induced dissociation (CID) and nano ESI ionisation in positive mode. The mass to charge was identified with a PASEF-MSMS scan mode for a mass range from 100 to 1700 m/z (Guzmán-Fierro et al. 2024). Peptide identification was done with the software tool PEAKS Studio (Bioinformatics Solutions, Canada) and compared to the private genomic database of the strain *Microbacterium* sp. BR1 provided by Dr. Boris Kolvenbach from the Institute for Ecopreneurship, University of Applied Sciences and Arts (Northwestern Switzerland).

The peptides were analysed with a label-free semi-quantification approach based on the spectral counting method and the Spec value (Zhang et al. 2013). Spec values considered the relative abundance of the enzymes linked to SMX metabolism by *Microbacterium* sp. BR1 (Sad cluster and DHPS). Additional bioinformatic analysis included processing the Gene Ontology (GO) categories of Biological Process (BP) with the Unipept Desktop 3.0 software (Verschaffelt et al. 2021) to investigate the effect of the tested SMX concentrations on diverse functional proteins. GO containing less than 2 peptides were not considered for the analysis (Zhao and Lin 2010).

## Results and discussion

### Biotransformation of SMX by *Microbacterium* sp. BR1

It has been reported that strain BR1 can mineralise up to 60% of the parent SMX when provided as the sole source of carbon and energy at environmentally relevant concentrations (25 µg L^−1^ down to 0.1 µg L^−1^) (Lopez Gordillo et al. 2024). In the present study, analytical measurements of the reaction media were used to investigate the biotransformation of SMX at a concentration range comparable to that reported in this mineralisation study.

**Fig. 1:**
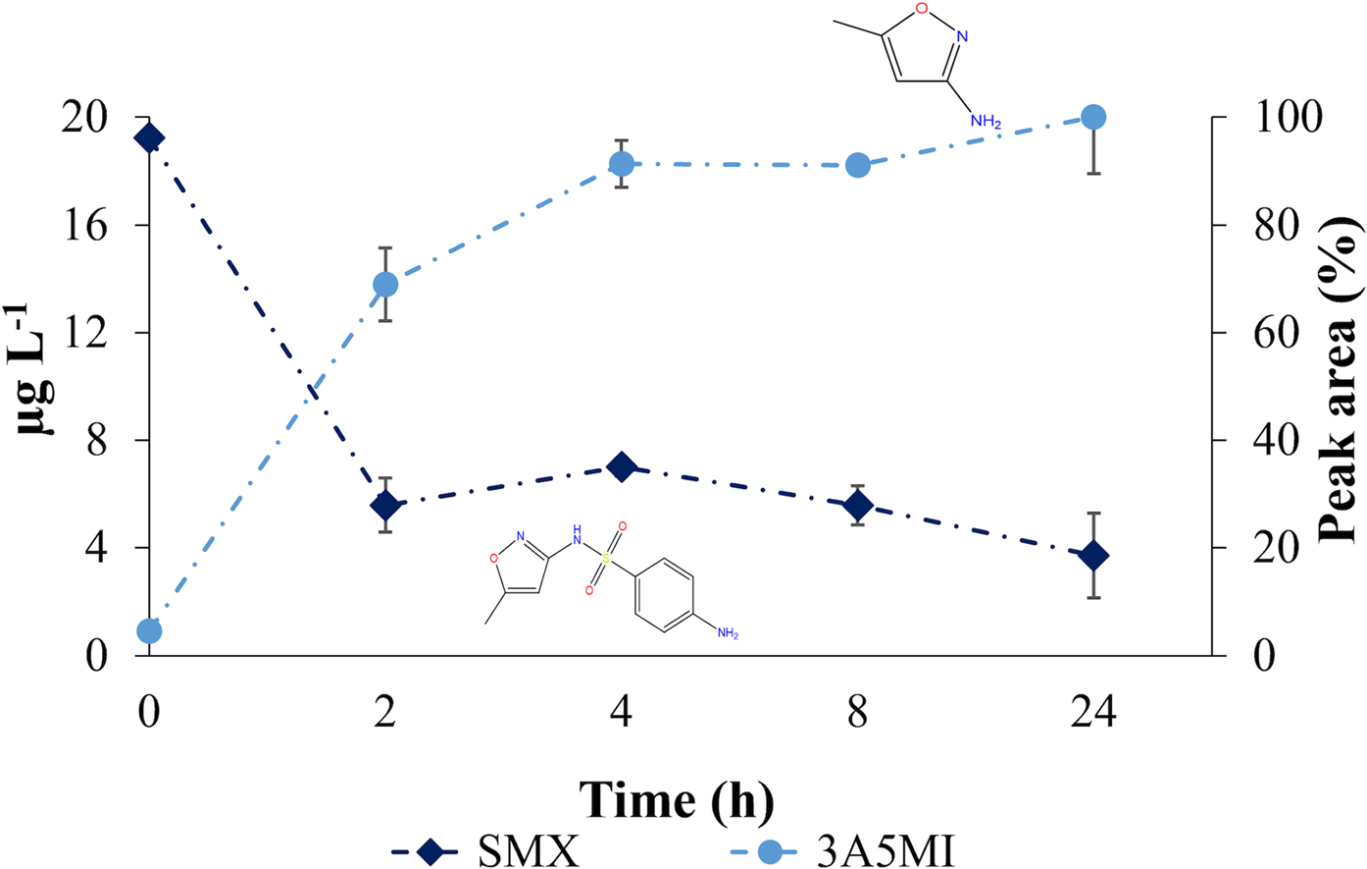
Evolution of SMX (diamond) and 3A5MI (circle) during biotransformation test with an initial concentration of 20 µg L^−1^. The relative chromatographic peak area on the right axis belongs only to 3A5MI whereas the concentration of SMX is given on the left axis. Depicted values are means of triplicates with their standard deviations. SMX = sulfamethoxazole; 3A5MI = 3-amino-5-methylisoxazole

The results of the supernatants confirm the idea that depletion of parent SMX is coupled to the production and accumulation of the metabolite 3A5MI in both the 20µg L^−1^ and 12 µg L^−1^ tests (Fig. 1 and online resource Fig. S2 accordingly).

The initial catabolic ipso-hydroxylation step of the parent SMX led to a pronounced drop in its concentration (5.59 µg SMX L^−1^) already by 2 h. After that, the residual SMX remained constant over time. The model fitting on SMX depletion indicated that a concentration of 5.42 µg L^−1^ was the turning point for the plateau, which started after 2 h. This plateau time matches the one from radiolabelled experiments performed at similarly low SMX concentrations of 25 µg L⁻^1^, 12.5 µg L^−1^ and 2.5 µg L^−1^ (Online resource Figure S3) (Lopez Gordillo et al. 2024). The results shown in Fig. 1 and online resource Fig. S2 are also in close agreement with previous investigations performed at a much higher concentration of 2.5 × 10^4^ µg SMX L^−1^ (Ricken et al. 2013), which suggests that the catabolic activity of strain BR1 is comparable over a wide range of SMX concentrations from µg L^−1^ up to mg L^−1^.

Environmental factors that may account for the degradation of antibiotics include adsorption, hydrolysis and photolysis (Yang et al. 2021). The contribution of these factors to the observed decrease of SMX can be disregarded based on the abiotic controls, where 95% of SMX was quantified after 24 h.

**Fig. 2:**
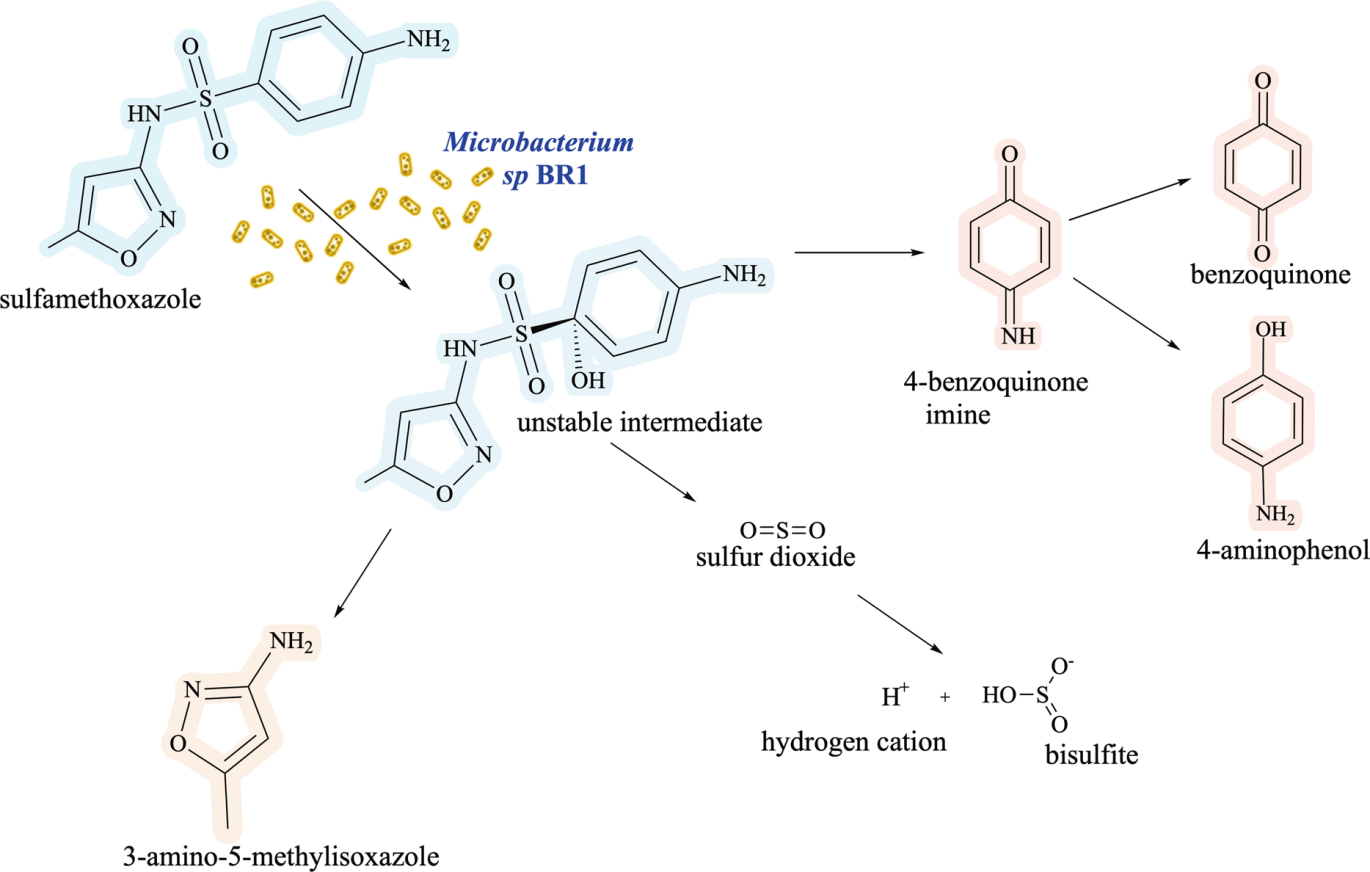
Ipso-hydroxylation has been described as the first step in SMX catabolism by strain BR1. After this step, the hydroxylated SMX molecule is split into 3-amino-5-methylisoxazole (abbreviated in the text as 3A5MI), sulfur dioxide (Ricken et al. 2013) and 4-benzoquinone imine. The solid arrows indicate the pathway reported in literature (Ricken et al. 2015, 2017). Modified from the original scheme with license CC BY (Ricken et al. 2013, 2017). SMX = sulfamethoxazole

The results of 3A5MI in this study together with hydroquinone detected in the analogous study (Lopez Gordillo et al. 2024) indicate that the commencement of the catabolic pathway of SMX by strain BR1 and some of the expected initial metabolites (Fig. 2) are likely similar to those described at 2.5 × 10^4^ µg SMX L⁻^1^ and 2.53 × 10^5^ µg SMX L^−1^ (Ricken et al. 2015, 2017). The catalytic flavin monooxygenase (SadA) together with the flavin reductase (SadC) are responsible for the initial attack of sulfonamide molecules, resulting in the release of 4-benzoquinone imine (BQI), 3A5MI and sulfur dioxide. In the catabolic pathway reported for strain BR1, BQI is split into benzoquinone and 4-aminophenol (Ricken et al. 2015, 2017).

The plateau of residual SMX in the presence of an acclimated bacterial strain over time, regardless of the different initial SMX concentrations, prompted investigation of the proteome profile to further understand the reasons behind the incomplete biotransformation and incomplete mineralisation (60%) (Lopez Gordillo et al. 2024) of the parent SMX.

### Proteome expression of *Microbacterium* sp. BR1 throughout SMX biotransformation

**Fig. 3:**
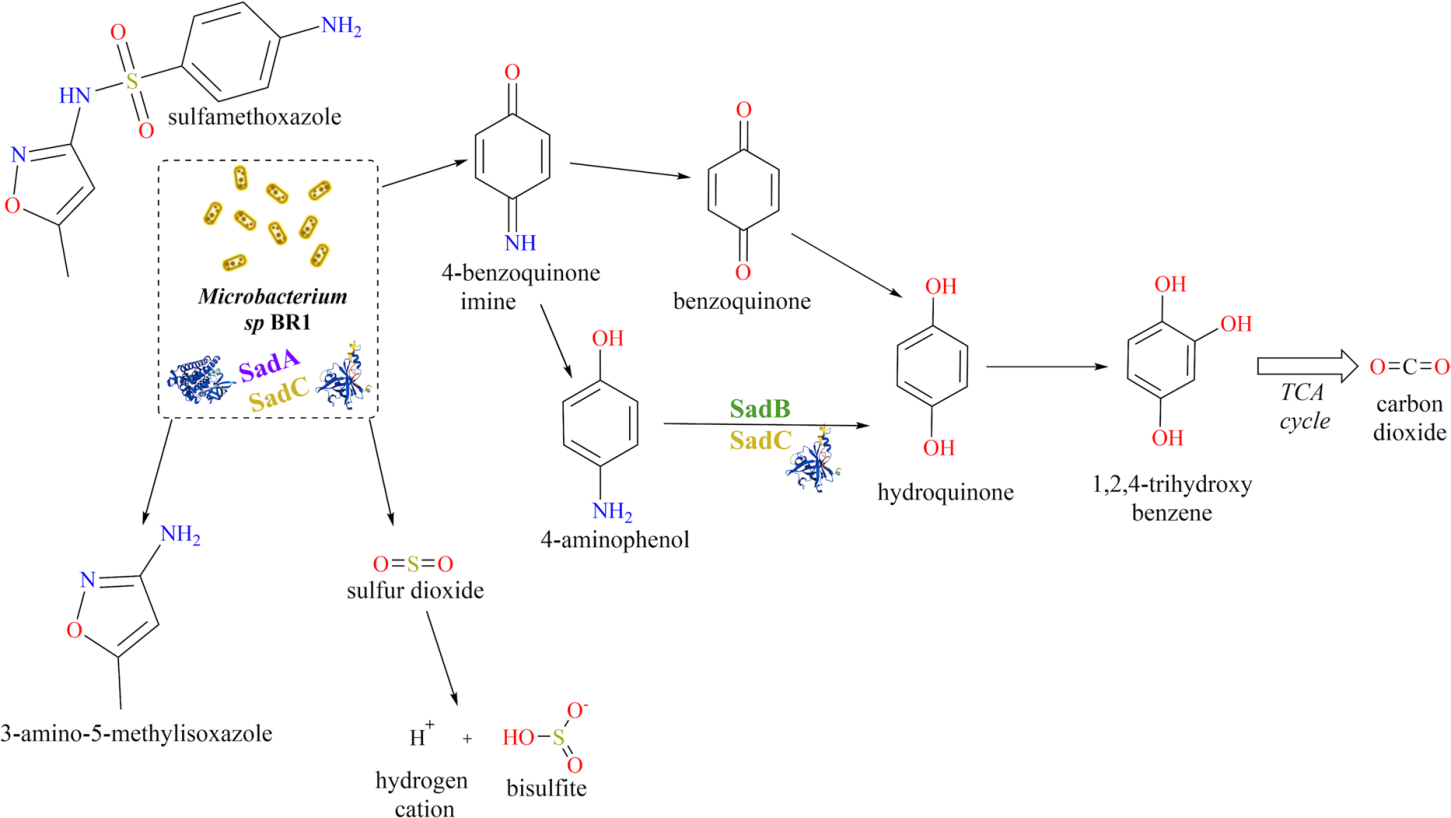
Reported metabolic pathway of the antibiotic sulfamethoxazole by strain BR1 (Ricken et al. 2013, 2015, 2017). The key enzyme cluster SadABC is displayed in the corresponding catabolic step. Metabolic pathway modified from the original scheme with license CC BY (Ricken et al. 2017)

SMX biodegradation studies (Ricken et al. 2017; Reis et al. 2019) have demonstrated that the catabolism of sulfonamides relies on the enzymes encoded in the SadABC cluster, which have been designated as sulfonamide degrading enzymes. After the initial step facilitated by SadAC, the production of 4-benzoquinone imine is the first step along the path towards mineralisation and concomitant biomass production (Fig. 3). The 4-aminophenol that is formed is then further transformed into 1,2,4-trihydroxybenzene by the flavoprotein monooxygenase SadB alongside SadC, which provides the necessary reduced flavin mononucleotide (FMN) (Ricken et al. 2017). The occurrence of this pathway during the degradation of SMX by strain BR1 has been verified by the production of CO_2_ and hydroquinone at concentrations from 0.1 µg SMX L^−1^ to 25 µg SMX L^−1^ (Lopez Gordillo et al. 2024), which encompass the concentrations of 20 µg SMX L^−1^ and 12 µg SMX L^−1^ tested in the present study. The pathway producing 3A5MI has been described as a dead end for strain BR1.

**Fig. 4:**
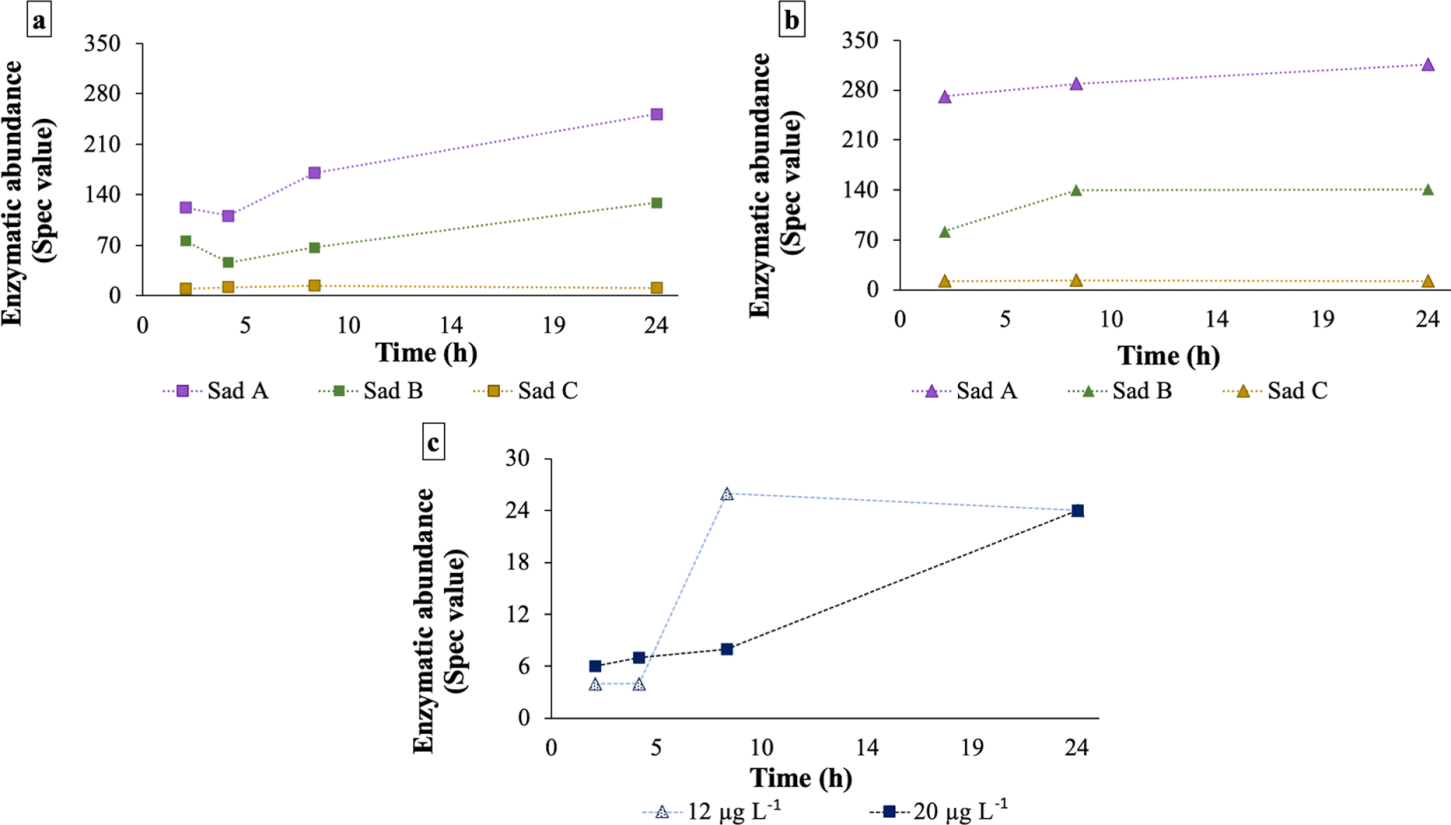
Relative abundance of the SadABC enzymes cluster over time in the tests with different initial SMX concentrations: **a)** 20 µg L^−1^ and **b)** 12 µg L^−1^. Panel **c)** depicts the relative abundance of sulfonamide resistance Sul1 for the two SMX concentrations as determined by the proteomic methodology in Sect. "Proteome analysis". Panel **b**) excluded one timepoint at 4 h identified as an outlier. SMX = sulfamethoxazole

To better understand the impediments to achieving complete metabolism of SMX, the expression of the different subunits of the SadABC protein complex was determined throughout the experiments (Online resources Tables S3-S6, and Fig. 4a-b). The catabolic activity of strain BR1 was triggered in all the tests, which manifested itself as an increase in the abundance of SadA and SadB over time in those experiments with 12 and 20 µg SMX L^−1^. Particularly noteworthy was that this triggering effect was observed even at a nominal SMX concentration below the analytical LOQ of 0.5 µg L^−1^ (Online resource Figure S4), which suggests that a similar catabolic activity occurs at very low SMX concentrations. Unlike the other Sad complex units, SadC expression was constant for all investigated concentrations (Fig. 4).

SMX acts as a competitive inhibitor of the enzyme DHPS, which produces the folate precursor necessary for the bacteria to reproduce (Baran et al. 2011; Revuelta et al. 2018). Some resistance mechanisms that allow bacteria to withstand this effect of sulfonamides include target modification (i.e., gene mutations) related to DHPS (*fol*P and *sul*1-*sul*4) and multidrug efflux systems (Sköld 2000; Blair et al. 2014; Sánchez and Martínez 2015; Sánchez-Osuna et al. 2018). The sulfonamide-resistant DHPS gene, *sul*, encodes for variations of this enzyme with low affinity to sulfonamides. Different sulfonamide degrading strains (e.g. *Actinobacteria* or *Micrococcaceae*) with known genome sequences contain *sad* and *sul* genetic clusters, which has led to the hypothesis that sulfonamide degradation might depend on sulfonamide resistance (Wu et al. 2023). In these experiments, the expression of Sul was detected for all test concentrations (Fig. 4c). At 20 µg L^−1^, there was a clear increase in expression over time. In contrast, at 12 µg L^−1^ there was a plateau. The fact that Sad and Sul followed similar expression trends at the different concentrations tested may represent biochemical evidence that further strengthens the hypothesis of sulfonamide resistance and degradation associated with this strain.

Moreover, the *sad* cluster has been described as a conserved genetic element with potential to be mobilized due to its suggested location within a putative composite transposon (Kim et al. 2019) or in a potential plasmid (Reis et al. 2019). However, the potential transmission of sulfonamide degradation genes (*sad*) together with the mobile sulfonamide resistance genes *sul* and *fol*P remains under discussion (Nunes et al. 2020). Recently, the presence of both genes *(sad* and *sul)* has been found via a BLAST search in all the *Actinobacteria* that efficiently degrade sulfonamides, including *Microbacterium* sp. BR1 (Wu et al. 2023)*.* This finding supports the idea that the expression of Sad and Sul in Fig. 4 may be linked. However, further genomic studies assessing SMX degradation of mutants with deleted *sul* or investigating the co-localization of *sad* and *sul* would be necessary to fully confirm their genetical linkage in the strain BR1.

**Fig. 5:**
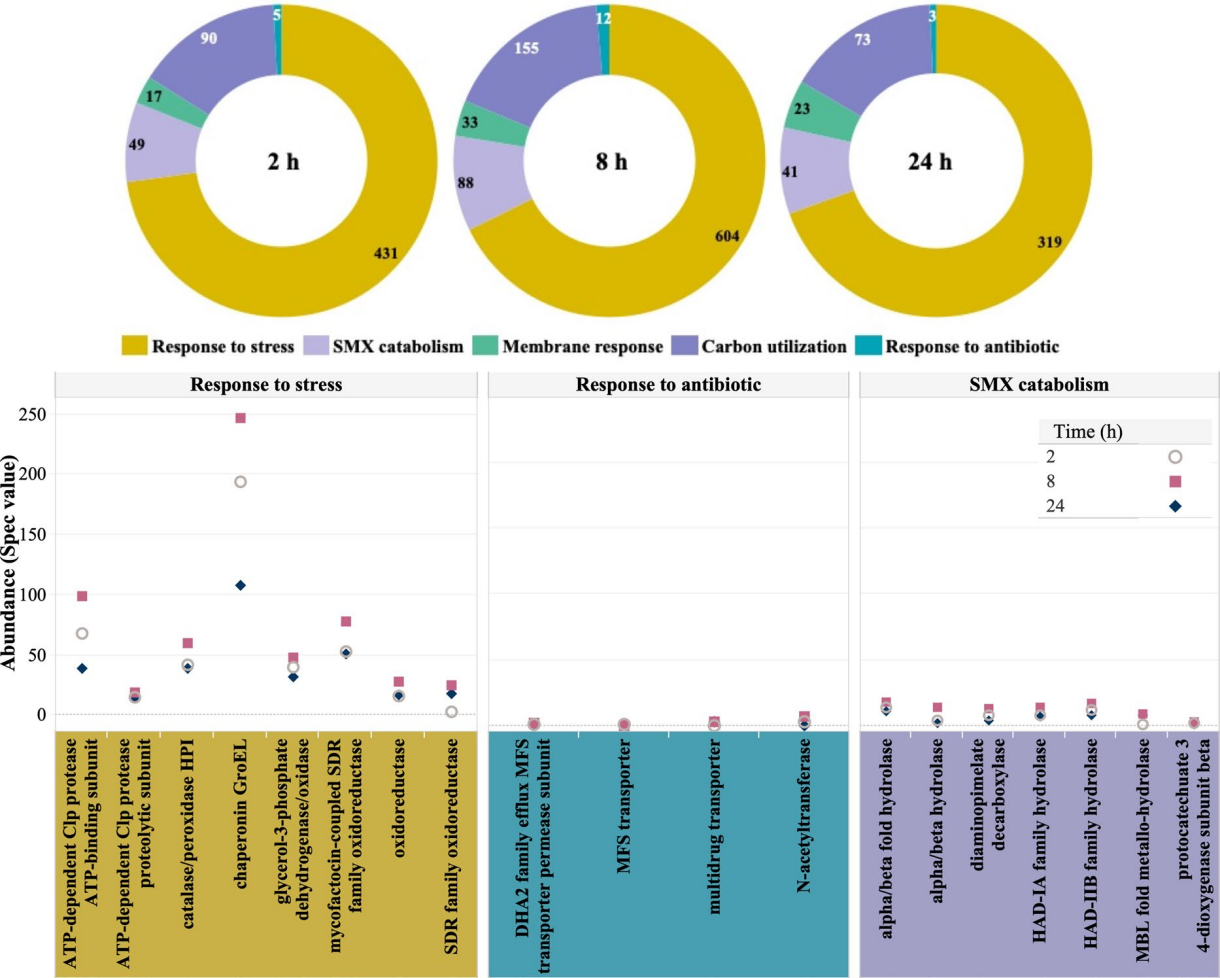
Change in relative abundance of proteins regulating SMX degradation in the test of 12 µg sulfamethoxazole L^−1^. For simplicity, proteins are grouped and their abundance (Spec values) displayed in donuts graphs (up) over time. Time series graphs (bottom) show changes on the relative abundance of the proteins from three representative groups: response to stress, response to antibiotic and SMX catabolism

Figure 5 depicts further analysis of the proteome profile of strain BR1, which aimed to investigate additional factors related to SMX degradation. Protein groups considered response to stress, catabolism of SMX, response to antibiotics and membrane response. From those, proteins related to stress were the most abundant over time, with an increase of proteases, oxidoreductases, peroxidases and chaperonins until 8 h, followed by a decrease at 24 h. This trend supports the result of most of SMX biotransformation occurring at 2 h and could correlate with the further catabolism of metabolites from the BQI pathway occurring until 8 h, when SMX mineralization by strain BR1 reaches a plateau, as previously described in Lopez Gordillo et al. (2024). Stress levels decreased by half by 24 h.

Interestingly, hydrolases dominated in the group of enzymes involved in SMX catabolism, with a higher group abundance (88) at 8 h. This is in line with any continued inactivation of the antibiotic (Zhang et al. 2024). Efflux pumps and transporters may respond to antibiotics by expelling them and their metabolites from the cell, whereas N-acetyltransferases acetylate SMX (Kagaya et al. 2012). Their low relative abundance indicates that the incomplete degradation of SMX and metabolites was not due to their constant extrusion from the cell. In fact, the low abundance of transporters favours intracellular diffusion to counteract low substrate concentrations (Kundu et al. 2019). Lastly, the sustained abundance in carbon utilization is attributed to proteins involved in the tricarboxylic acid cycle, which appoints for an active bacterial metabolism over the test.

**Fig. 6:**
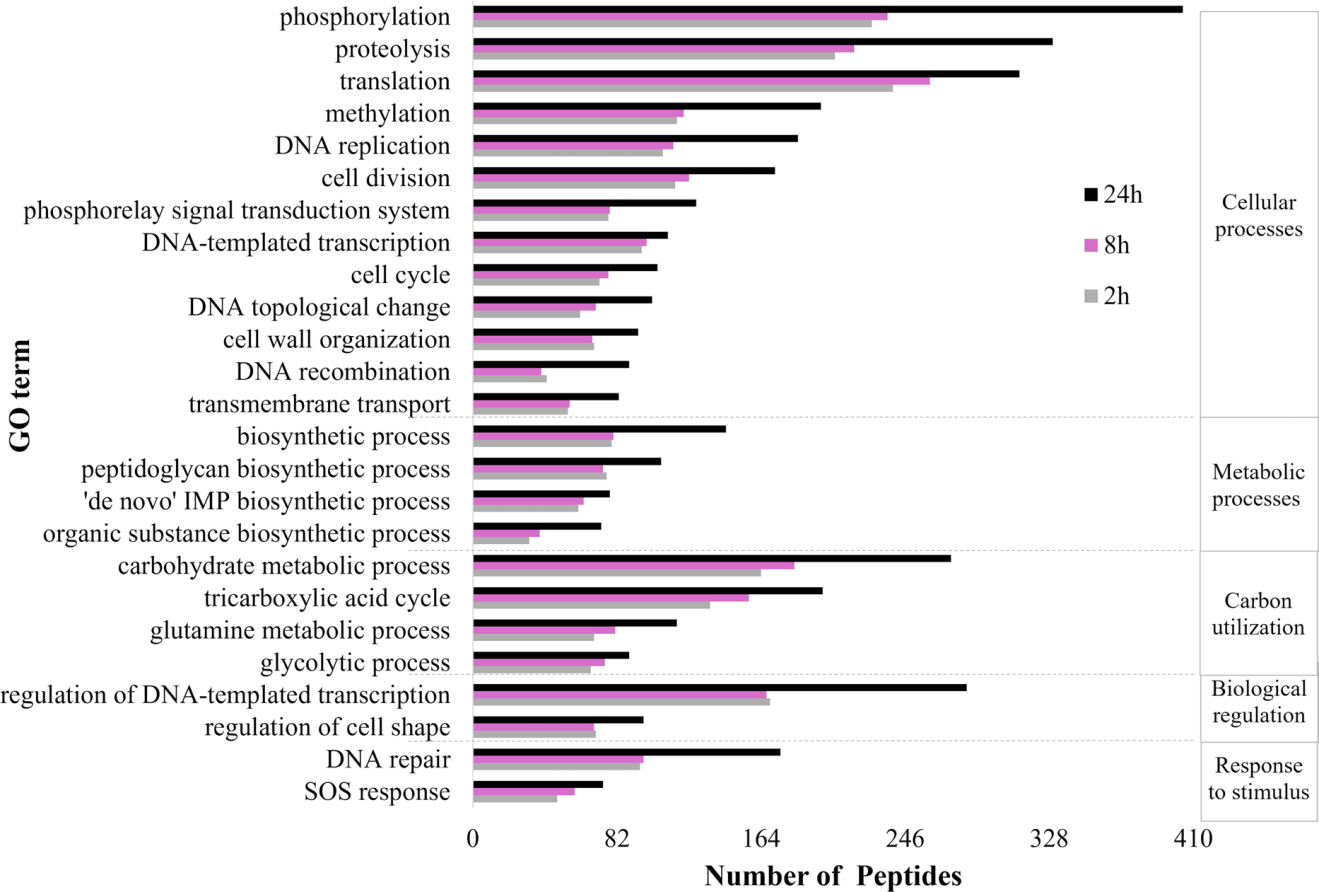
Change in peptide abundance from selected 25 biological processes (BP) in the experiments with 12 µg sulfamethoxazole L^−1^. Displayed Gene Ontology terms (GO) had the highest number of peptides over the test. For clarity, BP are grouped in categories (Carbon et al. 2009) and delimited with dotted lines

Research on the abundance of functional proteins allowed any changes in the general bacterial metabolism and stress due to exposure to SMX acting as sole source of carbon and energy to be investigated. For this, the peptides detected in our samples from the 12 µg SMX L^−1^ experiments were categorised according to the Gene Ontology classification of biological processes (Carbon et al. 2009; Verschaffelt et al. 2021) (Online resources Tables S7-S9). In general, the peptide abundance increased over time for processes related to cellular maintenance, such as cell division, cell cycle, phosphorylation, translation, or DNA replication (Fig. 6). Moreover, the peptide abundance in carbohydrate metabolism, the tricarboxylic acid cycle and biosynthetic processes also increased, confirming an active bacterial metabolism until the end of the test despite showing some signs of stress (Online resource Figure S5). Similar trends on the peptide abundance were observed in samples from the 20 µg SMX L^−1^ test (Online resource Figure S6).

An increase in the turbidity of the reaction media and the cell pellet size was observed over the 24 h, which indicates that SMX served as a carbon source. Bacterial growth is feasible considering that strain BR1 grows on the aniline moiety of SMX (Ricken et al. 2013). These observations together with the trends of peptides mentioned above, indicate that strain BR1 cells were still metabolically active. Therefore, biotransformation of the parent compound and transformation products must have stopped due to other reasons.

**Fig. 7:**
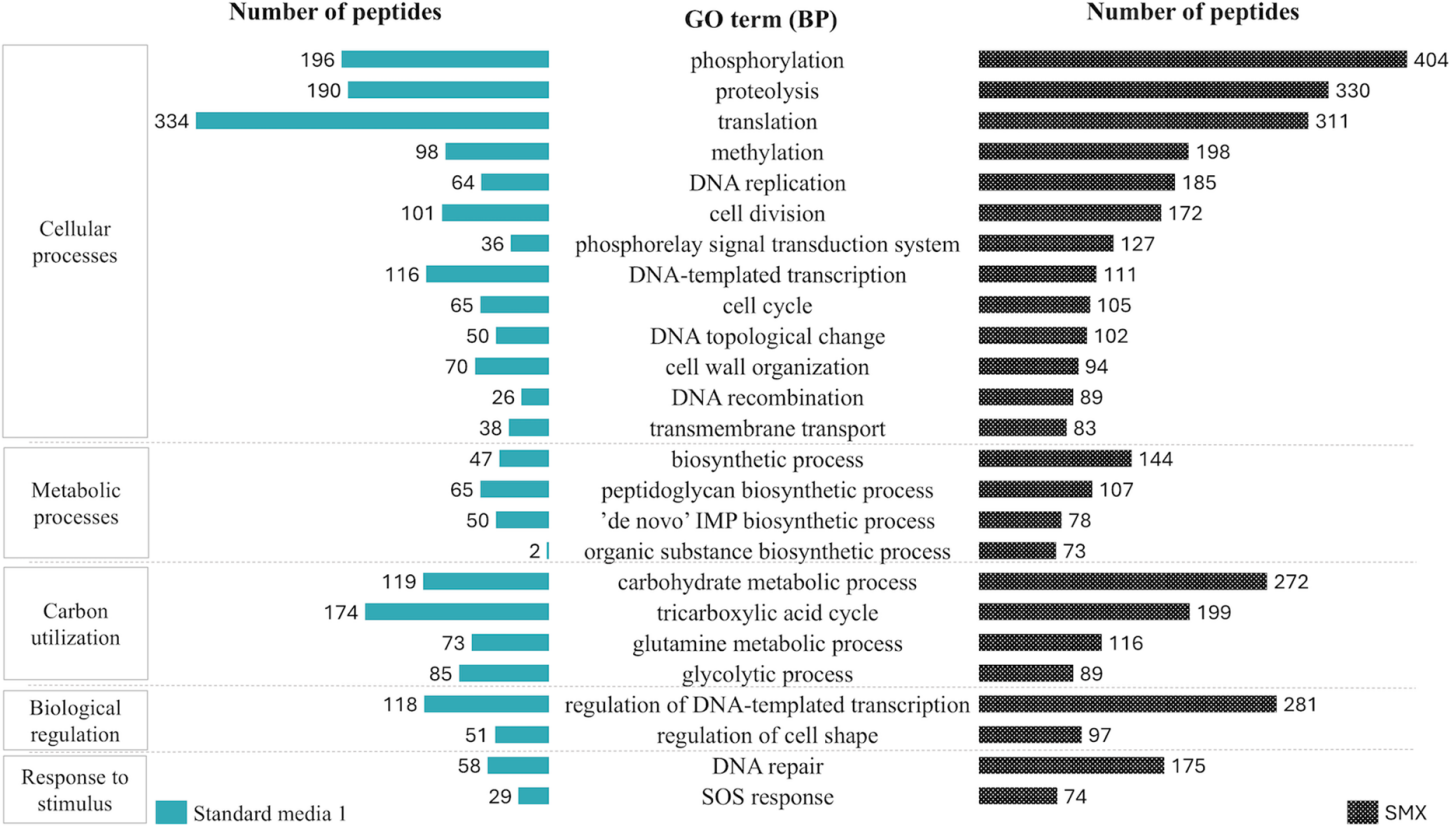
Contrast in peptide abundance of Top 25 biological processes (BP) found in *Microbacterium* sp. BR1 in a culture grown solely with complex media versus the end of biotransformation test with 12 µg SMX L^−1^ (24 h). BP are grouped in categories (Carbon et al. 2009) and delimited with dotted lines for clarity. GO = Gene Ontology

Interestingly, an increase in peptides related to the regulation of cell shape and cell wall organisation (Fig. 7) occurred over time, which could be attributed to morphological changes in the cells as a mechanism of antibiotic survival. Previous reports in the literature have pointed to changes in bacterial morphology due to exposure to antibiotics (Cushnie et al. 2016). These changes can be attributed to the exposure to SMX and not to carbon starvation or culture aging, since strain BR1 cultured in complex media without SMX presented a lower peptide abundance related to morphological changes.

Enzymes previously found in different microorganisms which enable aromatic ipso-substitutions and decrease the susceptibility towards antibiotics include laccases (Chivukula and Renganathan 1995), versatile peroxidases (VP) (Davila-Vazquez et al. 2005), 4-sulphobenzoate 3,4-dioxygenase (PSB dioxygenase system) (Locher et al. 1991), monooxygenases (CYP3A4, CYP3A5, CYP2D6*1) (Nakamura et al. 2011) or the flavoprotein monooxygenase 6-hydroxy-3-succinoyl-pyridine hydroxylase (HspB) (Tang et al. 2011). However, these enzymes are not encoded in the genome of strain BR1 (Wu et al. 2023) and, therefore, were not present.

### SMX persistence at low concentrations: accumulation and retro-inhibition by 3A5MI

As explained above, strain BR1 did not show limitations in its enzymatic activity or viability. Hence, these can be excluded as potential explanations for the observed incomplete removal of parent SMX during the biotransformation tests. Therefore, additional considerations related to the incomplete removal of SMX are discussed below.

Metabolites formed without SMX cleavage typically undergo back transformation to their parent SMX (Radke et al. 2009; Nödler et al. 2012; Chen and Xie 2018). Because 3A5MI is formed through direct metabolism by cleavage of the sulfonamide bond (S–N) in the SMX molecule, a back transformation to parent SMX cannot account for the partial removal of SMX by strain BR1.

As the relative abundance of catalytic enzymes (Sad) and the functional proteins related to cell growth, carbon utilisation and cell maintenance all increased (see Figs. 4 and 6), it can be assumed that the produced metabolites (including 3A5MI) are not toxic to the strain BR1. This lack of toxicity aligns with an analogous test, where strain BR1 was reported to be metabolically active and mineralised 0.5 µg SMX L^−1^ up to 48 h (Lopez Gordillo et al. 2024). A further study also supports the idea that 3A5MI lacks bacterial toxicity, where it was shown that a loss of the SMX bacteriostatic effect is attributed to its breakdown (Majewsky et al. 2014). A similar scenario was reported for *Achromobacter denitrificans* strain PR1, another SMX bacterial degrader that produces and accumulates 3A5MI as the primary metabolite lacking a toxic effect (Nguyen et al. 2017).

The fact that the metabolite 3A5MI is non-toxic does not rule out the possibility that it is less biodegradable—or even completely non-biodegradable—by strain BR1. The observed accumulation of 3A5MI in Fig. 1 and online resource Fig. S2 suggests that strain BR1 lacks the enzymatic machinery to degrade the 3A5MI produced. Although strain BR1 is a sulfonamide degrader, its degradation mechanism differs to that from pure bacterial strains capable of cleaving the SMX oxazole ring and 3A5MI (Mulla et al. 2018; Wang and Wang 2018; Yan et al. 2022). Recent studies showed an improved SMX and 3A5MI degradation in consortia or co-cultures of pure strains, when the enzymatic machineries complement each other (Qi et al. 2021, 2022; Chen et al. 2022; Wang et al. 2023).

SMX mineralization by strain BR1 has been enhanced in co-cultures with *Rhodococcus* sp. B2 (Bouju et al. 2012), which suggests that a complete removal of SMX and the accumulated 3A5MI could be feasible for artificial consortiums comprised of strain BR1 with strains capable of cleaving 3A5MI. Additional research could focus on exploring this possibility. However, within this study it has been shown that a pure culture of strain BR1 is unable to cleave the 3A5MI ring, resulting in the accumulation of this metabolite.

Inhibitory effects caused by the accumulation of additional metabolites derived from the downstream path of BQI seem unlikely for strain BR1 at both SMX test concentrations. Ricken et al. 2015 showed experimentally that strain BR1 degrades 4-aminophenol, benzoquinone, hydroquinone and 1,2,4-trihydroxybenzene at concentrations of 1.09 × 10^4^ µg L^−1^, 1.08 × 10^4^ µg L^−1^, 1.1 × 10^4^ µg L^−1^ and 1.26 × 10^4^ µg L^−1^ respectively, without accumulating intermediates causing oxidative stress. Therefore, any inhibition by metabolites points to the accumulated 3A5MI.

3A5MI accumulated in parallel to the decrease in the biotransformation rate of SMX, which became negligible from 2 h onwards (Fig. 1). The accumulation of 3A5MI has been experimentally linked to a delayed bacterial SMX biotransformation at concentrations of 1.52 × 10^5^ µg SMX L^−1^ (Reis et al. 2014), where a retro-inhibition by the end metabolite 3A5MI was attributed as the responsible mechanism. According to Fig. 1 and online resource Figure S2, this type of inhibition could also occur at low µg L^−1^ concentrations of SMX and might be a plausible explanation for the constant residual SMX. Further research could focus on exploring the retro-inhibitory activity of 3A5MI at low concentrations.

## Conclusions

The findings of this study help to unravel the biotransformation mechanism causing an incomplete biodegradation of SMX. The novelty of this investigation relies on exploring the ability of a bacterial strain to degrade concentrations at low µg L^−1^ concentrations, which are much lower than those reported in previous studies and more representative of the levels present in wastewater effluents. It was found that the biotransformation of SMX and the production of 3A5MI by strain BR1 are the same as that described at higher SMX concentrations (i.e., mg L^−1^). These results therefore demonstrate the suitability of an acclimated microorganism for degrading a wide range of concentrations of a target pollutant.

The combination of proteomics with quantification of the metabolites provided additional innovative insights (Zeng et al. 2022) into the SMX biodegradation pathway. Inactive SMX catalytic enzymes or non-viable cells could be ruled out as a cause of the incomplete degradation of the antibiotic by strain BR1. These findings may serve as a basis for future research to evaluate other potential causes of incomplete degradation of such OMP, including inhibition or thermodynamic equilibrium. Additionally, future research could focus on enhancing OMP degradation using mixed cultures of selected degraders with complementary degradation pathways, potentially improving bioremediation strategies for antibiotic contaminants in the environment.

## Environmental implications

Antibiotics like SMX are a hazard for the environment and health due to their potential to promote antibiotic resistance. Therefore, investigating the mechanisms behind their incomplete degradation is essential to achieve their full degradation and overcome their spread.

Several pure strains and mixed cultures have been used in biotransformation tests of SMX, but most of them do not combine quantification of the parent SMX and metabolites with the catalytic enzymes to investigate any incomplete biodegradation at trace concentrations as was done here. The results of this study therefore help to explain the incomplete biotransformation of SMX and similar organic micropollutants at low environmental concentrations.

## Supplementary Information

Below is the link to the electronic supplementary material.

Supplementary file1 (XLSX 519 KB)

Supplementary file2 (PDF 859 KB)

Supplementary file2 (PDF 1286 KB)

## Data Availability

The authors declare that the data supporting the findings of this study are available within the paper and its Supporting Information files.
