# Supplementary material for: Deciphering the constraints of pure bacterial strains for the complete catabolism of sulfamethoxazole: A proteomic and kinetic study
Source: Biodegradation. 2025 Nov 5;36(6):117. doi: 10.1007/s10532-025-10211-8 (PMC12589209; doi:10.1007/s10532-025-10211-8)
Supplement: Supplementary file 2 — Supplementary file2 (PDF 859 KB) [file 10532_2025_10211_MOESM2_ESM.pdf]

Supplementary Information

Deciphering the constraints of pure bacterial strains for the complete catabolism of sulfamethoxazole: A proteomic and kinetic study

Ana P. Lopez Gordillo [a,b*], Alba Trueba-Santiso [b], Kilian E.C. Smith [c], Andreas Schäffer [a] and Juan M. Lema [b]

[a] Institute for Environmental Research, RWTH Aachen University, Worringerweg 1, 52074 Aachen, Germany

[b] CRETUS, Department of Chemical Engineering, Universidade de Santiago de Compostela, 15782 Santiago de Compostela, Galicia, Spain.

[c] Environmental Chemistry, Department of Water, Environment, Construction and Safety, University of Applied Sciences Magdeburg-Stendal, Breitscheidstraße 2, 39114 Magdeburg, Germany.

[*]Corresponding author. E-mail address: ana.paulina.lopez@rwth-aachen.de

Supplementary data contains:

7 Pages

1 Text

2 Tables

6 Figures

Text S1 Analytical measurement of the parent SMX and 3A5MI. Mass spectrometry analyses were performed at the Mass Spectrometry and Proteomics Unit (Area of 201 Infrastructures) of the University of Santiago de Compostela. Defrosted supernatants from the biotransformation tests performed at concentrations of 20µg L^{-1} and 12 µg L^{-1} were analysed using a UHPLC ELUTE with a method including OLE up-concentration and a QTOF analyser. A 100 µL volume was injected and chromatographically separated on a C18 Intensity solo (Bruker) column. The technical specifications of the column were: 100 mm length, 2.1 µm inner diameter, 2 µm particle size and a pore size of 100 Å. For the elution, the mobile phase consisted of A) water + formic acid (FA) 0.1% and B) methanol + FA 0.1% with a constant flow rate of 0.250 mL min^{-1} and a gradient as described in Table S1. A capillary voltage of 4500 V was set as the ion source voltage, and the collision energy comprises 20-50 eV. The analysed mass range was from 50 to 750 m/z. with broadband collision-induced dissociation (bbCID) as a data-independent acquisition. A calibration curve prepared in PBS with the SMX and 3A5MI was used for the quantification of both compounds. The ionization of 3A5MI was not optimal at various stages of the run, only allowing the analysis of relative units (peak areas). The relative peak areas of 3A5MI were calculated considering the peak area at 24 h as 100%. Therefore, the proportions of 3A5MI at each sampling point are expressed relative to the peak area of 3A5MI at 24 h.

The specific m/z monitored corresponded to 99.055 for 3A5MI with a retention time (RT) of 3.16 minutes and 254.059 for SMX at RT=3.96 minutes. RT windows comprised 3.16 - 3.17 minutes and 3.95 - 3.98 minutes correspondingly. The respective limits of quantification (LOQ) were 0.1 µg L^{-1} for 3A5MI and 0.5 µg L^{-1} for SMX.

Table S1 Solvent gradient of the mobile phases used for the analysis of sulfamethoxazole and 3-amino-5-methylisoxazole.

Time (min)	%A	%B
**0**	95	5
**0.4**	95	95
**0.5**	75	25
**4**	25	75
**6**	0	100
**12**	0	100
**12**	95	5
**15**	95	5

%A= % H_2O+ FA 0.1%; **%B=** % Methanol + FA 0.1%.

Table S2 Protein concentration in extracted pellets of *Microbacterium* sp. BR1 quantified with the BCA test.

Test concentration	Sampling time (h)	Protein ($\mu g\ mL^{-1}$)*
**12 µg L^{-1}**	2	955
	4	851
	8	590
	24	878
**20 µg L^{-1}**	2	299
	4	415
	8	940
	24	1067

All the samples were resuspended in molecular grade water.

*For MS, the injected volume was adjusted per concentration to analyse comparable extracted proteins.

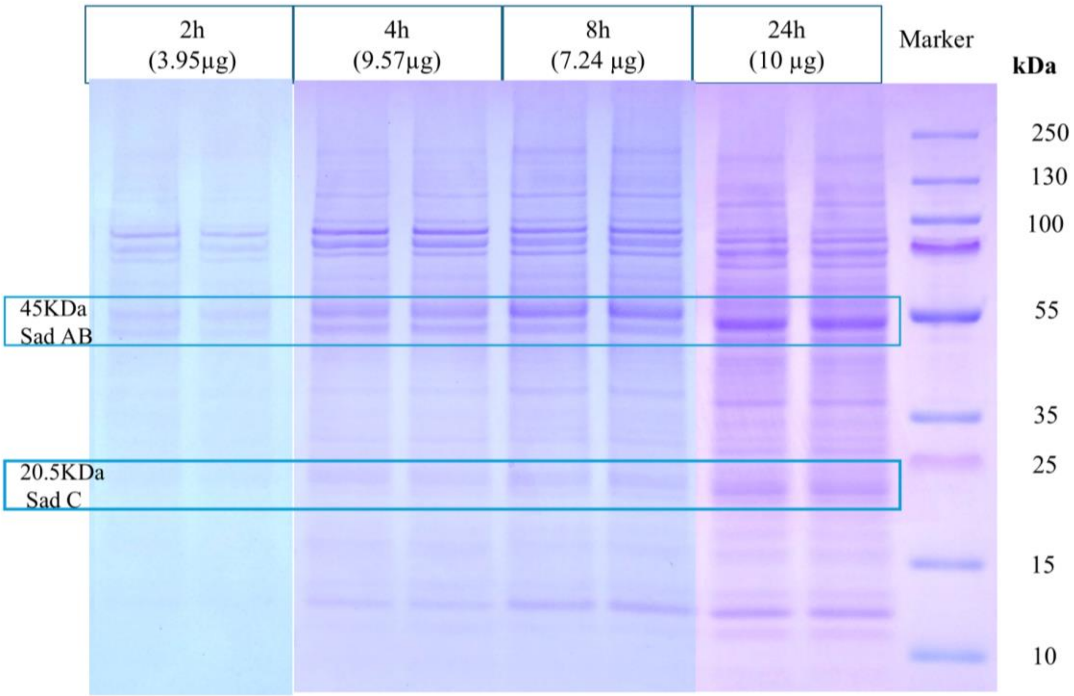

Figure S1 Bis-Tris Nu PAGE gel (4 – 12%) from the test 12 µg SMX L^{-1} stained with Coomassie after an electrophoresis run. The amount of protein extract loaded per lane appears in brackets. Duplicate lanes were loaded per sampling point. The *Marker* on the right column serve as reference for the bands of the samples. Bands aligned to where the Sad cluster enzymes would be retained are enclosed with a frame. SMX=sulfamethoxazole

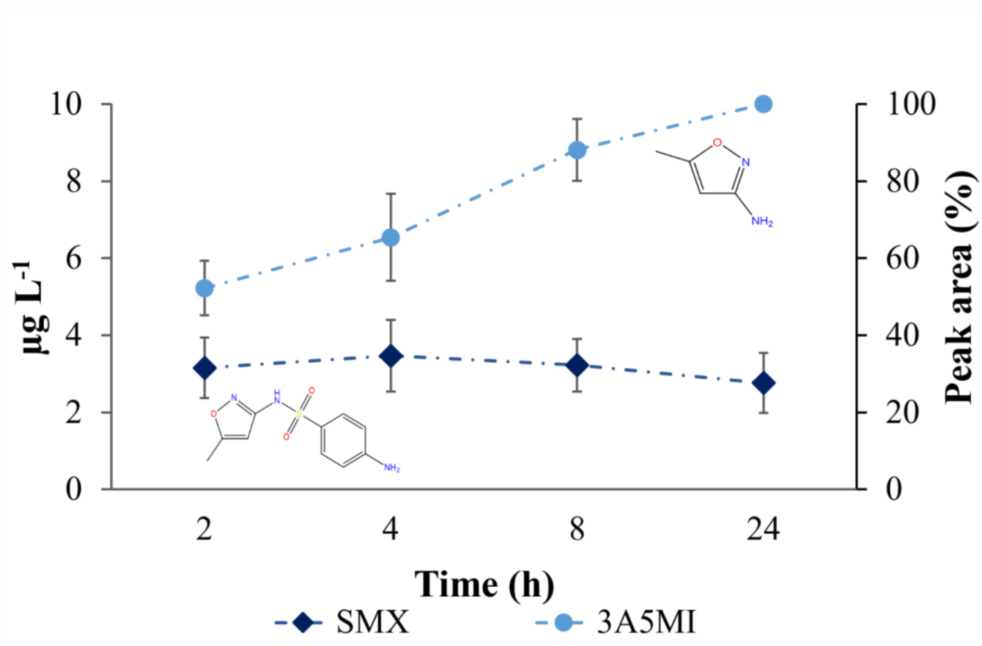

Figure S2 Evolution of SMX (diamond) and 3A5MI (circle) during biotransformation test with an initial concentration of 12 µg L^{-1}. The relative chromatographic peak area on the right axis refers only to 3A5MI whereas the concentration of SMX is given on the left axis. Depicted values are means of triplicates with their standard deviations. SMX = sulfamethoxazole; 3A5MI = 3-amino-5-methylisoxazole

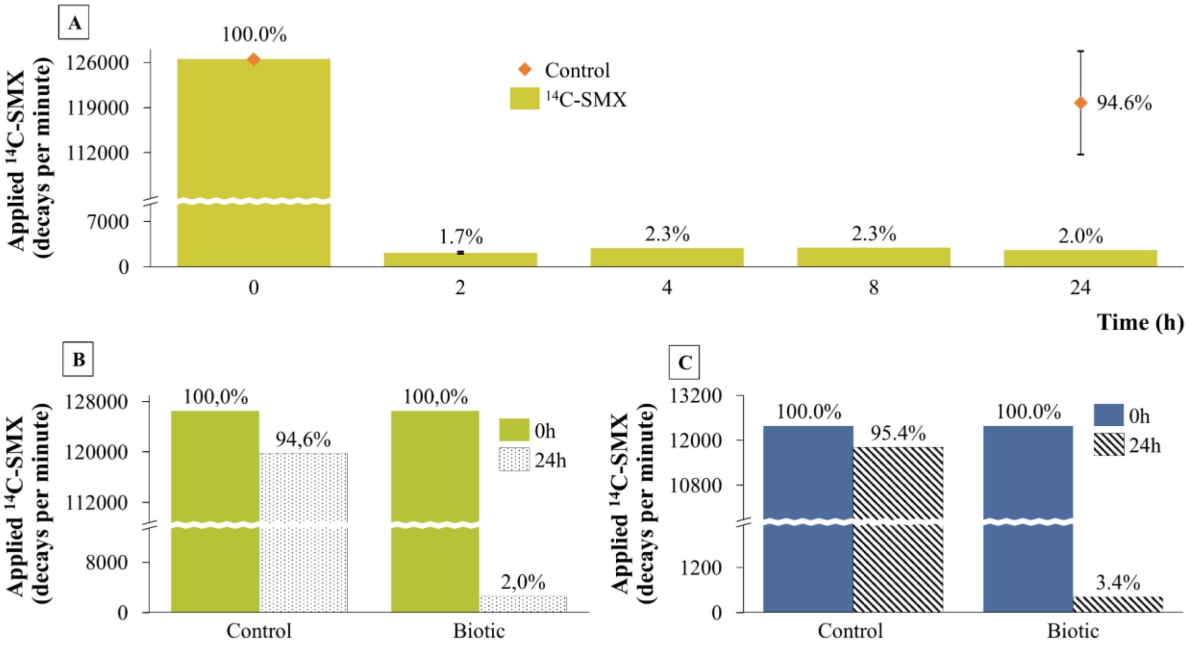

Figure S3 Comparison of the biotransformation of two concentrations of ^{14}C-SMX by *Microbacterium* sp. BR1. A) Time series biotransformation of 25 µg ^{14}C-SMX L^{-1}. The control (diamond) was measured only at the beginning and at the end of the test. B) Biotransformed fraction (shaded bars) of 25 µg ^{14}C-SMX L^{-1} after 24 h. C)

Biotransformed fraction (shaded bars) of 2.5 µg ^{14}C-SMX L^{-1} after 24 h. Controls comprised autoclaved (inactive) bacteria. Figure generated from data obtained in an analogous study (Lopez Gordillo et al., 2024).

A similar depletion of parent SMX occurred in both tests with a 10 fold concentration difference, where ca. 2 % of the initial parent SMX was found in biotic reactors contrasting to approximately 95 % found in the control by the end of the test.

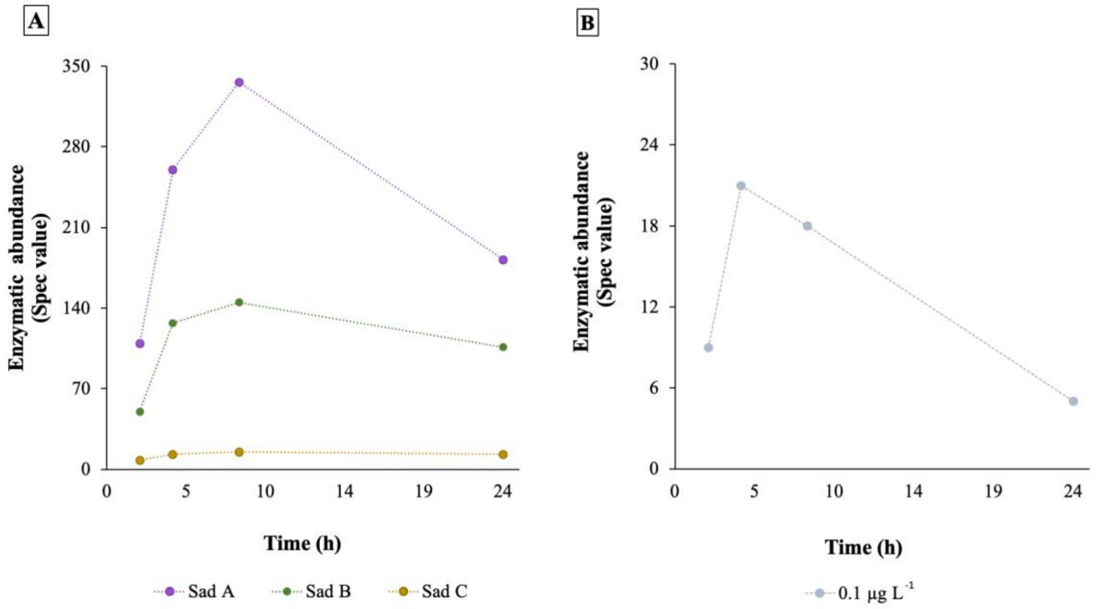

Figure S4 Relative abundance of enzymes involved in the catabolism of SMX. Panel A shows the SadABC enzymes cluster over time in a test with an initial SMX nominal concentration of 0.1 µg L^{-1}. Panel B depicts the relative abundance of sulfonamide resistance Sul1. SMX = sulfamethoxazole

Enzymes of the Sad cluster and Sul initially increased, followed by a decrease. This drop might be associated with a depletion of the parent molecule as reported for SMX biotransformation (Figure S3). An incomplete degradation of SMX was similarly confirmed in radiolabelled experiments with SMX mineralisation and biotransformation being similar for concentrations ranging from 25 µg L^{-1} down to 0.1 µg L^{-1}, see Figure 3 and Figure 4 in Lopez Gordillo et al., 2024.

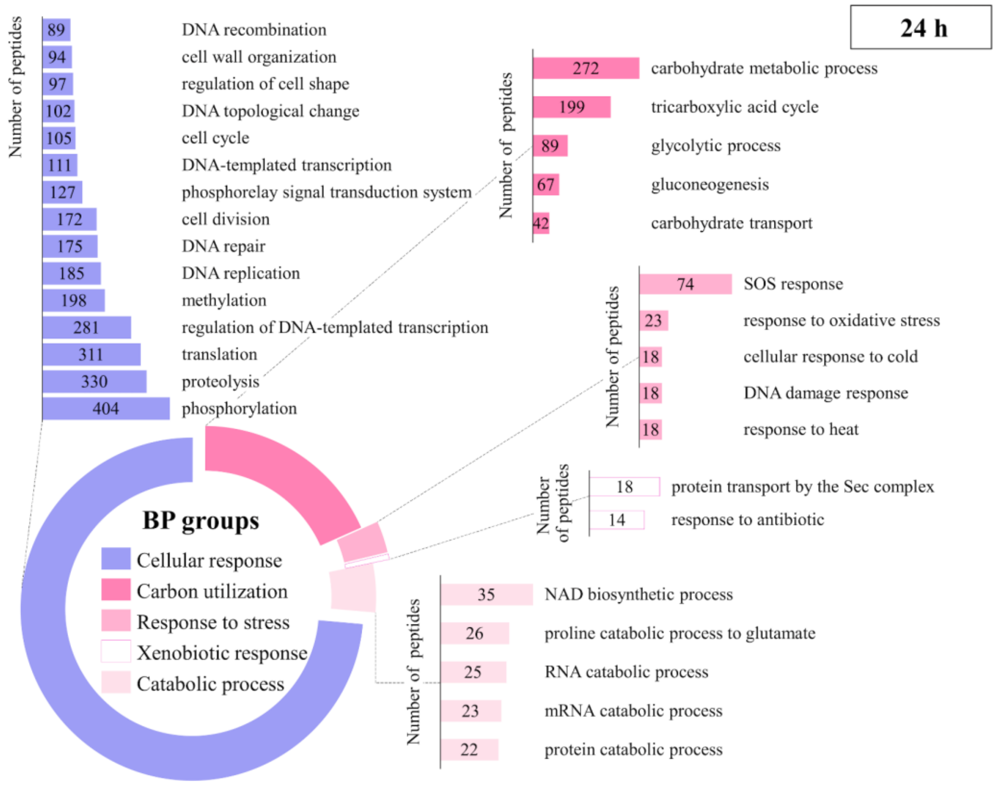

Figure S5 Peptide abundance of top Biological processes (BP) at 24 h in the test of 12 μg SMX L^{-1}. BP are grouped in five main categories to assess diverse bacterial reactions during biotransformation of SMX. SMX=sulfamethoxazole

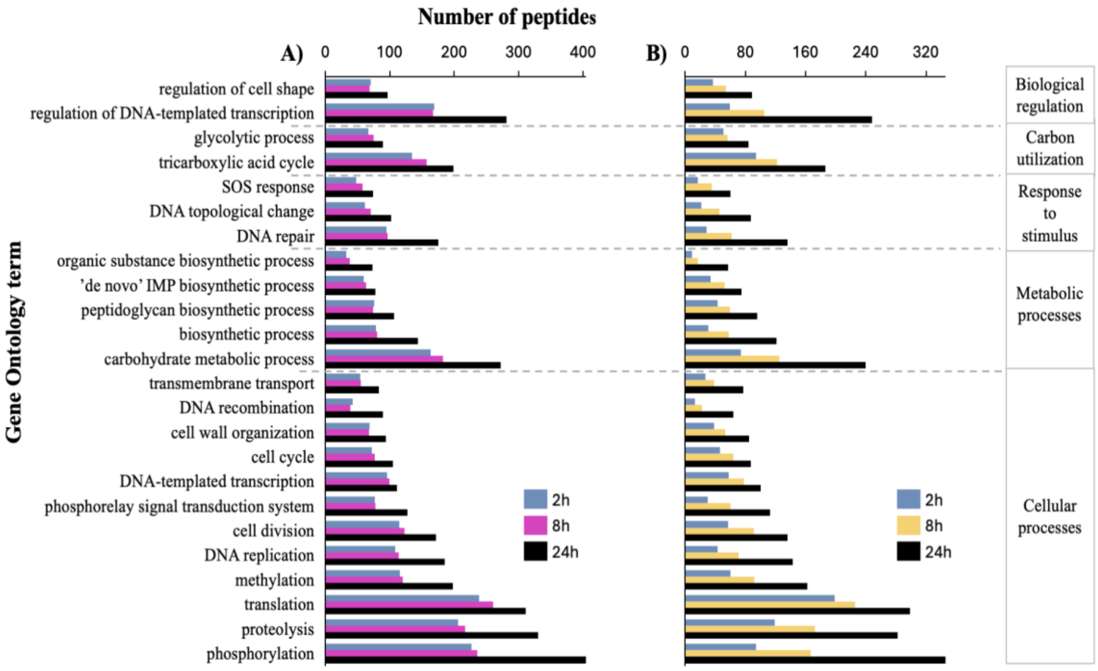

Figure S6 Top 24 common biological processes for both SMX biotransformation tests. Panel A shows the peptide abundance from the 12 μg SMX L^{-1} test. Panel B depicts the

relative peptide abundance from the 20 µg SMX L^{-1} test. The terms are grouped in categories (Carbon et al. 2009) and delimited with dotted lines. SMX = sulfamethoxazole

References

Lopez Gordillo, A.P., Trueba-Santiso, A., Lema, J.M., Schäffer, A., Smith, K.E.C., 2024. Sulfamethoxazole is Metabolized and Mineralized at Extremely Low Concentrations. Environ Sci Technol 58, 9723–9730. https://doi.org/10.1021/acs.est.4c02191

Carbon S, Ireland A, Mungall CJ, et al (2009) AmiGO: online access to ontology and annotation data. Bioinformatics 25:288–289. https://doi.org/10.1093/bioinformatics/btn615
